# Interspecies comparison of the early transcriptomic changes associated with hepatitis B virus exposure in human and macaque immune cell populations

**DOI:** 10.3389/fcimb.2023.1248782

**Published:** 2023-09-01

**Authors:** Armando Andres Roca Suarez, Séverine Planel, Xavier Grand, Céline Couturier, Trang Tran, Fabrice Porcheray, Jérémie Becker, Frédéric Reynier, Ana Delgado, Elodie Cascales, Loïc Peyrot, Andrea Tamellini, Adrien Saliou, Céline Elie, Chloé Baum, Bao Quoc Vuong, Barbara Testoni, Pierre Roques, Fabien Zoulim, Uzma Hasan, Isabelle Chemin

**Affiliations:** ^1^ INSERM U1052, CNRS UMR-5286, Cancer Research Center of Lyon (CRCL), Lyon, France; ^2^ University of Lyon, Université Claude-Bernard (UCBL), Lyon, France; ^3^ Hepatology Institute of Lyon, Lyon, France; ^4^ BIOASTER, Institut de Recherche Technologique, Lyon, France; ^5^ Department of Biology, The City College of New York, New York, NY, United States; ^6^ The Graduate Center, The City University of New York, New York, NY, United States; ^7^ CEA, Institut François Jacob, Fontenay-aux-Roses, France; ^8^ Inserm, U1184, Fontenay-aux-Roses and Université Paris-Saclay, Orsay, France; ^9^ Institut Pasteur de Guinée, Conakry, Guinea; ^10^ Department of Hepatology, Croix Rousse Hospital, Hospices Civils de Lyon, Lyon, France; ^11^ INSERM U1111, Centre International de Recherche en Infectiologie (CIRI), Lyon, France

**Keywords:** HBV, PBMC, transcriptomics, immune response, macaque

## Abstract

**Background and aims:**

Hepatitis B virus (HBV) infection affects 300 million individuals worldwide, representing a major factor for the development of hepatic complications. Although existing antivirals are effective in suppressing replication, eradication of HBV is not achieved. Therefore, a multi-faceted approach involving antivirals and immunomodulatory agents is required. Non-human primates are widely used in pre-clinical studies due to their close evolutionary relationship to humans. Nonetheless, it is fundamental to identify the differences in immune response between humans and these models. Thus, we performed a transcriptomic characterization and interspecies comparison of the early immune responses to HBV in human and cynomolgus macaques.

**Methods:**

We characterized early transcriptomic changes in human and cynomolgus B cells, T cells, myeloid and plasmacytoid dendritic cells (pDC) exposed to HBV *ex vivo* for 2 hours. Differentially-expressed genes were further compared to the profiles of HBV-infected patients using publicly-available single-cell data.

**Results:**

HBV induced a wide variety of transcriptional changes in all cell types, with common genes between species representing only a small proportion. In particular, interferon gamma signaling was repressed in human pDCs. At the gene level, interferon gamma inducible protein 16 (*IFI16*) was upregulated in macaque pDCs, while downregulated in humans. Moreover, *IFI16* expression in pDCs from chronic HBV-infected patients anti-paralleled serum HBsAg levels.

**Conclusion:**

Our characterization of early transcriptomic changes induced by HBV in humans and cynomolgus macaques represents a useful resource for the identification of shared and divergent host responses, as well as potential immune targets against HBV.

## Introduction

Hepatitis B virus (HBV) infection affects close to 300 million individuals worldwide, representing one of the major etiological factors for the development of cirrhosis and hepatocellular carcinoma (Yuen et al., 2018). At the molecular level, the mechanism behind chronic hepatitis B (CHB) is based on persistence of the viral genome as an episomal structure referred to as covalently closed circular DNA (cccDNA), which remains in the nucleus as a viral reservoir and template for viral replication (Martinez et al., 2021). In addition, HBV sequences can be integrated into the human genome and generate hepatitis B surface antigen (HBsAg) (Zhao et al., 2020). High concentrations of circulating HBsAg are associated with a dysfunctional immune response against HBV *via* a wide variety of mechanisms, which include the impairment of innate immune cells such as dendritic cells, monocytes, macrophages and natural killer (NK) cells, as well as HBV-specific T and B cells (Op den Brouw et al., 2009; Hong and Bertoletti, 2017; Faure-Dupuy et al., 2018; Tout et al., 2018; Bertoletti and Kennedy, 2019; Maini and Burton, 2019; Iannacone and Guidotti, 2022). These alterations do not exclusively result from the direct action of HBV on a particular cell type but can also stem indirectly from a dysregulated crosstalk between immune populations. For instance, it has been reported that HBsAg induces the generation of immunosuppressive monocytes, which are characterized by the expression of programmed death-ligand 1 (PD-L1) and major histocompatibility complex, class I, E (HLA-E). These HBV-induced monocytes educate NK cells to secrete higher levels of interleukin 10 (IL-10) and decrease the production of interferon gamma (IFN-γ), resulting in the inhibition of CD4^+^ and CD8^+^ T cells (Li et al., 2018).

Although nucleos(t)ide analogues are highly efficient at suppressing viral replication, HBV is never fully eliminated (EASL, 2017). Thus, these regimens require indefinite treatment to prevent the virological relapse that usually occurs after treatment discontinuation. This has led to a renewed interest to develop new curative strategies and combinations for HBV. In this context, it is believed that HBV cure will be a multi-layered combination approach of direct-acting antivirals and host-targeting agents in order to boost immune responses (Lim et al., 2023). However, the pre-clinical evaluation of immunomodulatory agents against HBV has been hampered by the lack of suitable *in vivo* models (Ortega-Prieto et al., 2019). Non-human primates, such as macaques, are widely used in virological studies due to their close evolutionary relationship to humans (Estes et al., 2018). Nonetheless, only low-titer or transient HBV replication has been observed in Mauritius cynomolgus macaques (*Macaca fascicularis*) (Dupinay et al., 2013). A similar situation has been reported in rhesus macaques (*Macaca mulatta*), in spite of expressing the human version of the HBV receptor sodium taurocholate co-transporting polypeptide (*hNTCP*) (Burwitz et al., 2017). Interestingly, it has recently been reported that viral clearance in macaques expressing *hNTCP* is mediated by an HBV-specific immune response and that immunosuppression leads to the development of a persistent infection (Biswas et al., 2022). Therefore, it is fundamental to have a solid understanding of the immunological differences between humans and non-human primate model organisms. This would allow not only to have a better grasp of the advantages and limitations of each model, but also the identification of potential targets for future clinical interventions. With this aim, we characterized the early transcriptomic changes and cytokine profiles induced by *ex vivo* exposure to HBV in human and cynomolgus macaque peripheral blood mononuclear cells (PBMCs). Thus, the present work represents a useful resource that allows the identification of shared and divergent transcriptional programs between both species, as well as the gene expression changes associated with each particular immune population of the same organism.

## Methods

### Blood sample collection and PBMC isolation

Human blood samples (*n*=6) were obtained from the French national blood service (Etablissement Français du Sang, Lyon, France). Mauritius cynomolgus blood samples (*n*=6) were provided by the platforms Bioprim and Cynbiose, in agreement with international guidelines (CITES n°FR1503100944-I). PBMCs were isolated using Ficoll-Paque PLUS (GE Healthcare). Samples were frozen in fetal bovine serum (Sigma Aldrich) supplemented with 10% DMSO (Sigma Aldrich) and stored in liquid nitrogen until the day of the experiments.

### *Ex vivo* HBV exposure

PBMCs of human or macaque origin were thawed, re-suspended in RPMI-1640 (Thermo Fisher Scientific) and evaluated for cell number and viability. For each condition, 15x10^6^ PBMCs were incubated for 2h at 37°C with 5% CO_2_ in RPMI-1640 culture medium ± HBV (genotype D, 100 viral genome equivalents/cell) or mock inoculum, which were produced as previously described (Tout et al., 2018). Supernatants were frozen for subsequent cytokine analyses.

### Cytokine profiling

IL-10, IFN-γ and tumor necrosis factor alpha (TNF-α) were analyzed in supernatants by Luminex array. Species-dedicated kits were used in order to ensure that antibodies did not cross-react. Custom Milliplex Non-Human Primate and Human Cytokine Magnetic Bead Panel (Millipore) were used according to the manufacturer’s instructions. All samples were analyzed using a Bio-Plex 200 reader and the Bio-Plex Manager Software (Bio-Rad).

### Cell sorting

PBMCs were stained with the antibody panel (**Supplementary Table 1**) for 15 min at RT, resuspended in D-PBS and immediately sorted into plasmacytoid dendritic cells (pDCs), myeloid dendritic cells (mDCs), T cells and B cells using a FACS Aria II (BD Biosciences) (**Supplementary Table 2**, **Supplementary Figure 1**).

### RNA extraction and bulk RNA sequencing (RNA-seq)

A minimum of 5000 sorted cells per sample were lysed in Trizol (Life Technologies) and RNA was extracted using Phasemaker™ Tubes Complete System (Thermo Fischer Scientific), according to the manufacturer’s instructions. A purification step was added after RNA extraction using the RNA Clean & Concentrator™-5 kit (Zymo Research). The SMART-Seq v4 Ultra Low Input RNA Kit for Sequencing (Takara Bio) was used for cDNA synthesis, according to the user manual recommendations. Purification of amplified cDNA was performed using the Agencourt AMPure XP kit (Beckman Coulter) and controlled on a 2100 Bioanalyzer using High Sensitivity DNA chips. Following cDNA quantification with the GloMax^®^ Multi-detection system (Promega), all samples were diluted to 30 pg/µL and 150 pg were used for Nextera XT fragmentation according to the Nextera XT protocol. Libraries were diluted to 1 nM and denatured with 0.2 M NaOH at RT for 5 min. Subsequently, 0.2 M Tris-HCl pH 7 was added to ensure that NaOH was fully hydrolyzed in the final solution. Denatured libraries were diluted to 20 pM with pre-chilled hybridization buffer. Paired-end sequencing was performed on a NextSeq 500 sequencer using the NextSeq 500 High Output v2 kit (150 cycles, Illumina).

### Bioinformatics analyses

*Bulk RNA-seq:* Quality of the raw sequences was verified using FastQC. All samples presented a similar profile, with an average Phred quality score above 30. Reads were quantified using the quasi-mapping-based mode of Salmon (Patro et al., 2017). This step needed a preliminary indexing of reference transcripts, which was performed using the “salmon index” command with default setting k-mer size k=31. The references used were the Genome Reference Consortium Human Build 38 (hg38, Genbank accession GCA_000001405.15) for the human analysis and Macaca_fasicularis_5.0 (Genbank accession GCA_000364345.1) for the macaque analysis. Raw counts were processed using the DESeq2 package in R/Bioconductor (Love et al., 2014) (**Supplementary Table 3**). Pre-ranked gene set enrichment analysis (GSEA) was performed using GenePattern and the gene sets belonging to the Molecular Signatures Database (MSigDB) v7.5.

*Single-cell RNA sequencing (scRNA-seq):* PBMC transcriptomic and clinical data from healthy and HBV-infected patients (*n*=23) was obtained from the Gene Expression Omnibus (GEO) database accession GSE182159 (Zhang et al., 2023). Sample integration was performed using the *FindIntegrationAnchors* and *IntegrateData* functions of Seurat (v4.2) (Satija et al., 2015). Cell clustering was performed with the *FindClusters* function of Seurat using a resolution of 0.5 for the analysis, which resulted in 25 clusters. Marker genes for each of the clusters were identified with the *FindMarkers* function of Seurat and were employed to provide labels to 14 major cell types (**Supplementary Figure 2A**).

### Statistical analyses

Comparison of cytokine levels in the supernatant of HBV-stimulated and control samples was performed using a Wilcoxon matched-pairs test. Statistical tests were performed using GraphPad Prism software v9.4 (GraphPad Software).

Differential expression analysis between groups was performed using the Wald test in DESeq2. Gene expression changes were considered significant if presenting a false discovery rate (FDR)=<0.05 following correction for multiple testing using the Benjamini-Hochberg method.

## Results

### Short-term *ex vivo* HBV exposure induces transcriptomic changes associated with the immune response in human and macaque PBMC populations

**Figure 1:**
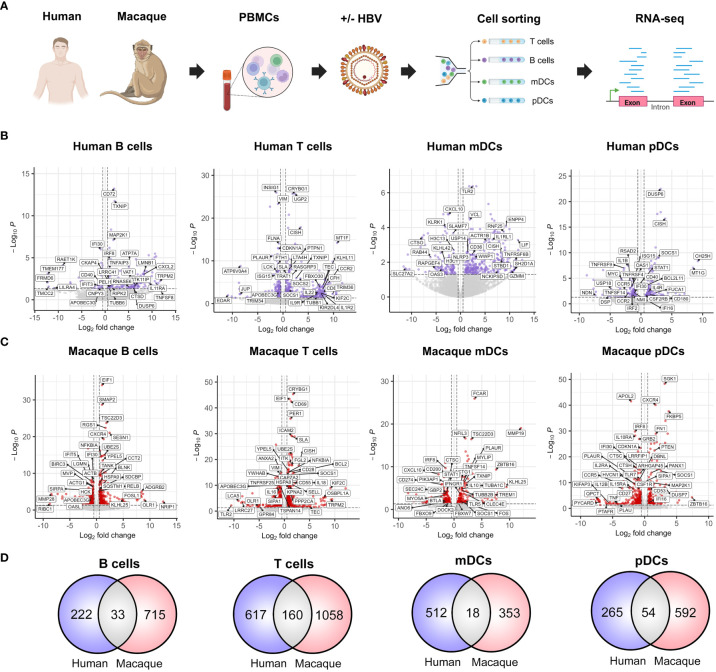
Interspecies comparison of the early immune responses to HBV in human and cynomolgus macaque PBMCs. **(A)** PBMC samples from HBV-negative human donors without HBV vaccination (*n*=6) and Mauritian cynomolgus macaques (*n*=6) were cultured *ex vivo ±* HBV (100 viral genome equivalents/cell, 2h). Cells were subsequently FACS-sorted into T cell, B cell, mDC and pDC populations. Paired-end sequencing was performed in order to obtain transcriptomic data for each cell type, species and condition. **(B, C)** Volcano plots depicting significantly modulated immune response genes (FDR=<0.05) in each of the **(B)** human and **(C)** macaque cell populations. **(D)** Number of individual genes modulated in response to the presence of HBV (FDR=<0.05, Wald test). Venn diagrams show human-specific (blue), macaque-specific (red) and common (gray) gene expression changes.

With the aim to perform a comprehensive characterization of the early transcriptomic changes induced by HBV in the peripheral immune compartment, we isolated PBMCs from HBV-negative donors without HBV vaccination (*n*=6) and Mauritian cynomolgus macaques (*n*=6), which were subsequently exposed to HBV *ex vivo* during a two-hour period. PBMCs were then sorted into T cell, B cell, mDC and pDC populations in order to perform bulk RNA-seq (**Figure 1A**). This allowed us to observe a wide variety of significantly (FDR=<0.05, Wald test) modulated genes induced by HBV exposure in each cell type from both species (**Figures 1B, C**). The total number of differentially-expressed genes in human and macaque immune populations were: 222 vs 715 in B cells, 617 vs 1058 in T cells, 512 vs 353 in mDCs and 265 vs 592 in pDCs, respectively (**Figure 1D**). Some of the expression changes common to humans and macaques included the upregulation of suppressor of cytokine signaling 1 (*SOCS1*), *SOCS2* and cytokine inducible SH2-containing protein (*CISH*) in T cells, which have been described to reflect their activation following antigen stimulation (Yu et al., 2003). In addition, we observed the downregulation of apolipoprotein B mRNA editing enzyme catalytic subunit 3G (*APOBEC3G*) in B and T cells. This is of particular relevance, as APOBEC3G has been reported to play a key role during the host immune response against HBV (Turelli et al., 2004). Nonetheless, common genes between both species represented only a small proportion of the total transcriptomic alterations in response to HBV, with the majority of modulated genes being specific to each species (**Figure 1D**). These results suggest that HBV is able to induce markedly different transcriptional profiles in human and macaque PBMCs early during the host immune response.

### *IFI16* expression is impaired in pDCs exposed to HBV *ex vivo* and persists in HBV-infected patients

**Figure 2:**
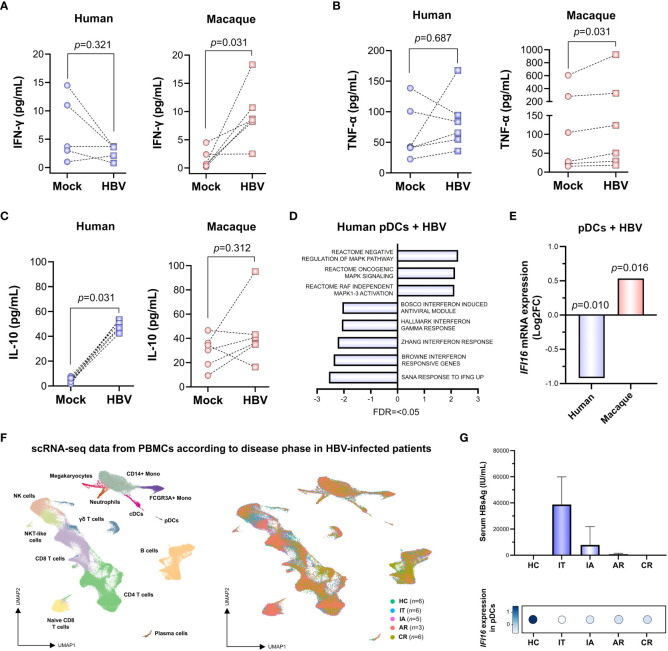
*IFI16* expression is impaired in pDCs exposed to HBV *ex vivo* and persists in HBV-infected patients with long duration. **(A–C)** Interferon gamma (IFN-γ), tumor necrosis factor alpha (TNF-α) and interleukin 10 (IL-10) levels in the supernatant of human and macaque PBMCs exposed *ex vivo* to HBV (Wilcoxon matched-pairs test). **(D)** GSEA showing a significant negative enrichment of IFN pathways in human pDCs exposed to HBV, in spite of an increased activity of pro-inflammatory MAPK signaling (FDR=<0.05). **(E)** Expression of *IFI16* is significantly downregulated in human pDCs and upregulated in macaque pDCs exposed to HBV *ex vivo* (FDR=<0.05, Wald test). **(F)** scRNA-seq data analysis depicting the multiple immune cell populations (left) identified in PBMCs from patients (*n*=23) at different phases of natural HBV infection with long duration (right), including immune tolerant (IT), immune active (IA), acute recovery (AR), chronic resolved (CR) and HBV-free healthy controls (HC) (GSE182159). **(G)** HBsAg serum levels (top) are inversely associated with the expression of *IFI16* in pDCs (bottom) according to disease phase.

In order to gain further insights into the mechanisms leading to this species-specific transcriptomic modulation, we quantified cytokine levels produced in response to HBV exposure. This allowed us to observe a significant increase (*p*=<0.05, Wilcoxon matched-pairs test) in the production of IFN-γ and TNF-α in macaque PBMCs exposed to the virus (**Figures 2A, B**). This stands in contrast to the increased levels of IL-10 in human PBMCs (**Figure 2C**), suggesting that HBV induces two different cytokine responses, a pro-inflammatory profile in simian and an anti-inflammatory one in humans. In particular, pathway analysis of the human pDC population showed that not only is there a lack of IFN-γ signaling induction, but that the response *via* this pathway is downregulated as a whole (**Figure 2D**). At the individual gene level, a clear example that illustrates this situation is interferon gamma inducible protein 16 (*IFI16*), a DNA sensor previously shown to inhibit HBV cccDNA transcriptional activity (Yang et al., 2020). Indeed, HBV stimulation induced the downregulation of *IFI16* in human pDCs, which is markedly different from its upregulation in macaque pDCs (**Figure 2E**). Furthermore, we wanted to explore if the impairment of *IFI16* could be observed *in vivo* at later stages of the disease. Therefore, we analyzed a recently available scRNA-seq PBMC dataset (Zhang et al., 2023), obtained from healthy controls (HC, *n*=6) or individuals at different stages of the disease, including immune tolerant (IT, *n*=6), immune active (IA, *n*=5), acute recovery (AR, *n*=3) and chronic recovery (CR, *n*=6) (**Figure 2F**, **Supplementary Figure 2A**). In this cohort, serum levels of HBsAg seem to be inversely associated with the expression of *IFI16* in the pDC population (**Figure 2G**). These results suggest that repression of *IFI16* takes place early during infection, showing the lowest levels at disease phases characterized by high viral load and progressively increasing with HBsAg loss. A similar repression was observed for additional components of the IFN-γ signaling pathway, such as N-Myc and STAT interactor (*NMI*) and interferon regulatory factor 2 (*IRF2*) (**Supplementary Figure 2B**).

## Discussion

In the present study, we aimed to characterize the gene expression changes taking place early after exposure to HBV in the peripheral immune compartment (**Figure 1A**), as these events represent the starting point for the development of more complex intra- and intercellular immune responses (Netea et al., 2019). Moreover, our comparison between human and macaque immune populations provides a global view of the common and divergent innate responses between both species (**Figure 1D**). Thus, representing a useful resource to improve our understanding of HBV animal models and the identification of pathways to activate antiviral immune responses and viral clearance.

The initial examination of transcriptomic profiles from human and macaque immune populations seems to indicate that macaque PBMCs present a stronger immune response to the contact with HBV particles, as compared to human PBMCs. This may indicate that macaques are more prone to mount immune responses to HBV infection. Indeed, we describe how macaque PBMCs exposed to HBV secrete IFN-γ and TNF-α, while human PBMCs show only an increased secretion of IL-10 (**Figures 2A–C**). This is a relevant observation, as it has recently been described that distinct T cell populations can be identified in HBV-infected patients based on their TNF-α/IFN-γ profile. In particular, successful differentiation of TNF-α-producing T cells into IFN-γ-producing T cells is associated with HBV clearance (Wang et al., 2020). Moreover, we identified the DNA sensor *IFI16* as an IFN pathway component that is downregulated in human pDCs exposed to HBV *ex vivo*. Interestingly, analysis of publicly available transcriptomic data from patients presenting a natural HBV infection with long duration showed that *IFI16* expression levels parallel HBsAg loss (**Figure 2G**). This is in line with previous reports describing how an increase in *IFI16* expression following peg-IFN treatment was associated with hepatitis B e antigen (HBeAg) seroconversion in CHB patients (Lu et al., 2021). Considering that *IFI16* levels increase following HBV clearance but do not seem to completely return to basal levels, it would be interesting to explore its potential epigenetic alteration. Indeed, this has been observed for components of the IFN-γ pathway in response to HBV infection (Lim et al., 2018).

In summary, our dataset serves as a valuable resource to gain a deeper understanding of immune responses taking place during HBV infection. This and similar studies could prove useful for the identification of potential targets against HBV.

## Data availability statement

The datasets presented in this study can be found in online repositories. The names of the repository/repositories and accession number(s) can be found below: https://www.ncbi.nlm.nih.gov/geo/query/acc.cgi?acc=GSE223073.

## Ethics statement

The studies involving humans were approved by French National Blood Service (Etablissement Français du Sang, Lyon, France). The studies were conducted in accordance with the local legislation and institutional requirements. The human samples used in this study were acquired from French National Blood Service (Etablissement Français du Sang, Lyon, France). Written informed consent for participation was not required from the participants or the participants’ legal guardians/next of kin in accordance with the national legislation and institutional requirements. The animal study was approved by international guidelines (CITES n°FR1503100944-I). The study was conducted in accordance with the local legislation and institutional requirements.

## Author contributions

Conceptualization: IC; Methodology: IC and UH; Data acquisition: SP, AT, FP, CC, AD, EC, LP, FR, AS, and CB; Formal analysis: AR, XG, JB, TT, CC, BV, UH, and IC; Writing – original draft: AR; Writing – review & editing: XG, BV, BT, PR, FZ, UH, and IC. All authors contributed to the article and approved the submitted version.
