# Supplementary material for: Interspecies comparison of the early transcriptomic changes associated with hepatitis B virus exposure in human and macaque immune cell populations
Source: Front Cell Infect Microbiol. 2023 Sep 1;13:1248782. doi: 10.3389/fcimb.2023.1248782 (PMC10505653; doi:10.3389/fcimb.2023.1248782)
Supplement: Supplementary file 1 [file DataSheet_1.docx]

***Supplementary Material***

Interspecies comparison of the early transcriptomic changes associated with hepatitis B virus exposure in human and macaque immune cell populations.

**Armando Andres Roca Suarez^1,2,3^, Séverine Planel^4^, Xavier Grand^1,2,3^, Céline Couturier^4^, Trang Tran^4^, Fabrice Porcheray^4^, Jérémie Becker^4^, Frédéric Reynier^4^, Ana Delgado^4^, Elodie Cascales^4^, Loïc Peyrot^4^, Andrea Tamellini^4^, Adrien Saliou^4^, Céline Elie^4^, Chloé Baum^4^, Bao Quoc Vuong^5,6^, Barbara Testoni^1,2,3^, Pierre Roques^7,8,9^, Fabien Zoulim^1,2,3,10^, Uzma Hasan^1,11^, Isabelle Chemin^1,2,3,^***

^1^INSERM U1052, CNRS UMR-5286, Cancer Research Center of Lyon (CRCL), Lyon, France.

^2^University of Lyon, Université Claude-Bernard (UCBL), Lyon, France.

^3^Hepatology Institute of Lyon, Lyon, France.

^4^BIOASTER, Institut de Recherche Technologique, 40 avenue Tony Garnier, 69007, Lyon, France.

^5^Department of Biology, The City College of New York, New York, NY, United States.

^6^The Graduate Center, The City University of New York, New York, NY, United States.

^7^CEA, Institut François Jacob, Fontenay-aux-Roses, France.

^8^Inserm, U1184, Fontenay-aux-Roses and Université Paris-Saclay, Orsay, France.

^9^Institut Pasteur de Guinée, Conakry, Guinea.

^10^Department of Hepatology, Croix Rousse Hospital, Hospices Civils de Lyon, Lyon, France.

^11^INSERM U1111, Centre International de Recherche en Infectiologie (CIRI), Lyon, France.

***Correspondence:**Isabelle Chemin
isabelle.chemin@inserm.fr

**Supplementary Table 1**…………………………………………………………..……………..……2

**Supplementary Table 2**…………………………………………………….……………….…..……3

**Supplementary Figure 1**...……………………………………………………………………………4

**Supplementary Figure 2**...……………………………………………………………………………5

| **Markers** | **Fluorochromes** | **Clone** | **Manufacturer** | **Reference** |
| --- | --- | --- | --- | --- |
| **CD66b** | PerCP Vio700 | TET2 | Miltenyi | 130-103-864 |
| **CD14** | PerCP Cy5.5 | M5E2 | Biolegend | 301823 |
| **CD3** | BV421 | SP34-2 | BD Biosciences | 562877 |
| **CD8** | BB515 | RPA-T8 | BD Biosciences | 564526 |
| **CD56** | FITC | NCAM16.2 | BD Biosciences | 345811 |
| **HLA-DR** | BV510 | L243 | Biolegend | 307646 |
| **CD123** | APC | 763 | BD Biosciences | 560087 |
| **CD11c** | PE | 3.9 | Biolegend | 301605 |
| **CD20** | PE Cy7 | L27 | BD Biosciences | 335828 |
| **Zombie NIR** | APC Cy7 |  | Biolegend | 423105 |

Supplementary Table 1. Antibody cocktail panel for the labeling of peripheral blood mononuclear cells (PBMCs) and their sorting into T cells, B cells, myeloid dendritic cells (mDCs) and plasmacytoid dendritic cells (pDCs).

| **Setting** | **70 µm** |
| --- | --- |
| **Sheath pressure** | 70 |
| **Amplitude** | 60 |
| **Frequency** | 87 |
| **1^st^ drop** | 150 |
| **Gap (upper limit)** | 6 (14) |
| **Attenuation** | Off |
| **Drop delay** | 47.00 |
| **Far left voltage** | 100 |
| **Left voltage** | 40 |
| **Right voltage** | 40 |
| **Far right voltage** | 100 |
| **Plate voltage** | 4,500 |
| **2^nd^ drop** | 20 |
| **3^rd^ drop** | 10 |
| **4^th^ drop** | 0 |

Supplementary Table 2. Sorting parameters for the isolation of T cells, B cells, mDCs and pDCs from human and macaque PBMCs. All experiments were performed on a FACS Aria II (BD Biosciences) using a 70 µm nozzle.


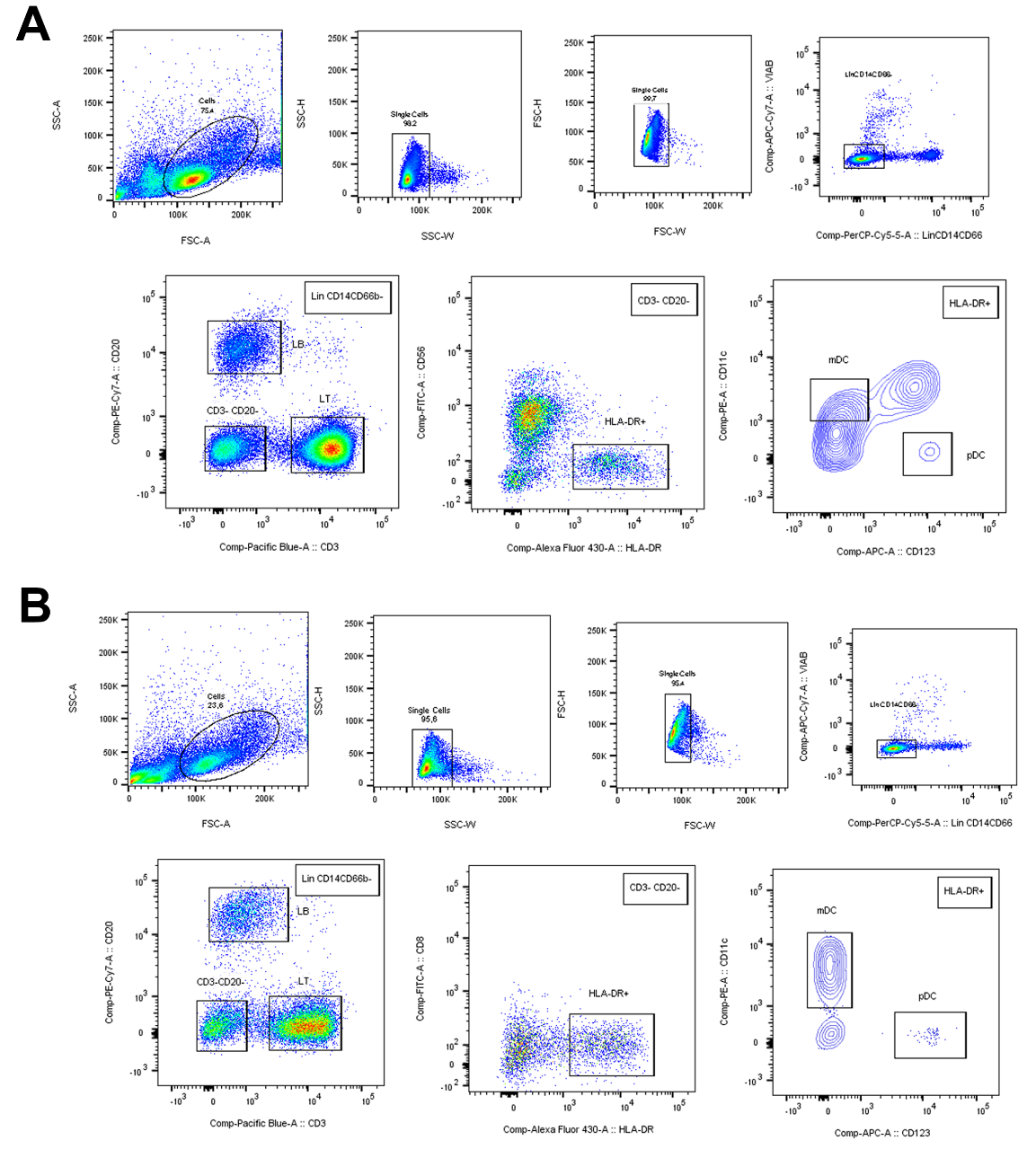


**Supplementary Figure 1.** Gating strategy for the sorting of human and cynomolgus macaque immune populations. CD14^-^CD66^-^ cell populations were used for the identification of T cells (CD3^+^) and B cells (CD20^+^). CD3^-^CD20^-^HLA-DR^+^ cells were further selected for the sorting of mDCs (CD11c^+^) and pDCs (CD123^+^). Representative samples are shown to depict the gating of (**A**) human and (**B**) macaque PBMCs.


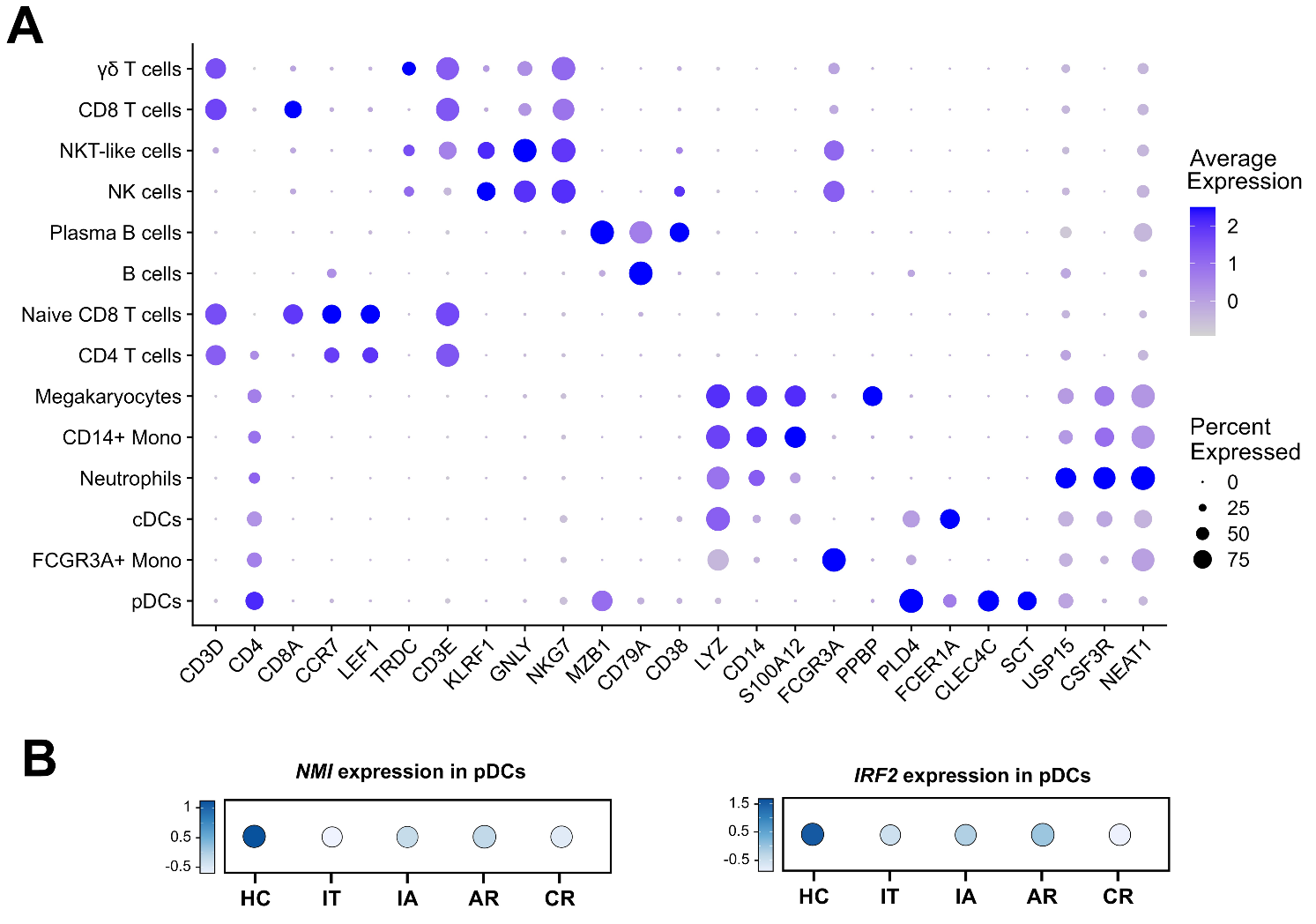


**Supplementary Figure 2.** (**A**) Dotplot showing the average expression levels and percentage of cells expressing canonical cell type markers in each of the 14 cell types identified in PBMC samples from HBV-infected patients. (**B**) Expression of N-Myc and STAT interactor (*NMI*) and interferon regulatory factor 2 (*IRF2*) in pDCs according to disease phase (GSE182159).
